# Serosurvey of anti-*Toxocara canis* antibodies in people experiencing homelessness and shelter workers from São Paulo, Brazil

**DOI:** 10.1186/s13071-022-05499-x

**Published:** 2022-10-17

**Authors:** Vamilton Alvares Santarém, Anahi Chechia do Couto, Susana Zevallos Lescano, William Henry Roldán, Ruana Renostro Delai, Rogério Giuffrida, Louise Bach Kmetiuk, Alexander Welker Biondo, Sriveny Dangoudoubiyam, Andrea Pires dos Santos

**Affiliations:** 1 Graduate College in Animal Sciences, University of Western São Paulo (UNOESTE), Presidente Prudente, SP 19050-920 Brazil; 2 Graduate College of Cellular and Molecular Biology, Federal University of Paraná (UFPR), Curitiba, PR 81531-970 Brazil; 3 Institute of Tropical Medicine of São Paulo, University of São Paulo, Butantã, SP 05403-000 Brazil; 4 Department of Veterinary Medicine, Federal University of Paraná (UFPR), Curitiba, PR 80035-050 Brazil; 5 Department of Comparative Pathobiology, College of Veterinary Medicine, Purdue University, West Lafayette, IN USA

**Keywords:** Seroprevalence, Toxocariasis, Zoonosis, Homeless

## Abstract

**Background:**

Despite being one of the most prevalent helminth parasitic zoonoses worldwide and particularly in socioeconomically vulnerable populations, toxocariasis remains to be fully investigated in persons experiencing homelessness. Accordingly, the present study has aimed to assess the seroprevalence and associated risk factors of *Toxocara* spp. exposure in persons experiencing homelessness and shelter workers from a day-shelter in São Paulo city, Brazil.

**Methods:**

Anti-*Toxocara* IgG antibodies were detected by enzyme-linked immunosorbent assay (ELISA). Univariable and multivariable logistic regression models were performed to assess the risks for toxocariasis.

**Results:**

Overall, anti-*Toxocara* IgG antibodies were detected in 89/194 (45.9%, 95% CI: 39.0–52.9%) persons experiencing homelessness, twice as high (OR = 2.2; 95% CI = 1.245–3.873; *P* = 0.0089) than the frequency of 22/79 (27.8%, 95% CI: 19.2–38.6) in shelter workers. College education was the only protective factor for *Toxocara* spp*.* exposure (OR: 0.23; *P* = 0.018) revealed by logistic regression.

**Conclusions:**

Although indicating a multifactorial origin of toxocariasis, the present study has assessed a highly vulnerable population with high disease risks and premature death. Thus, the living conditions of the homeless population have influenced the high prevalence of anti-*Toxocara* antibodies verified here compared with domiciled shelter workers. Despite being less exposed, shelter and other outdoor workers may present an occupational risk to toxocariasis. Future studies should establish whether such environmental exposure might occur in persons experiencing homelessness in other regions worldwide.

**Graphical Abstract:**

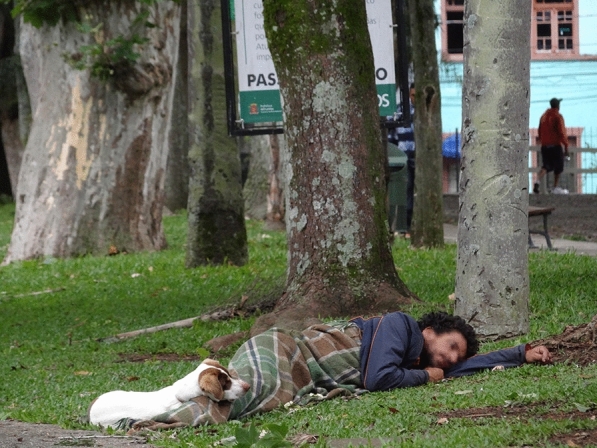

**Supplementary Information:**

The online version contains supplementary material available at 10.1186/s13071-022-05499-x.

## Background

Toxocariasis is considered one of the most frequent and relevant neglected parasitic zoonoses worldwide [1]*.* Toxocariasis is caused by the common roundworms *Toxocara canis* and *Toxocara cati*, whose definitive hosts are dogs and cats, respectively [2]. Humans become infected most commonly by accidental ingestion of embryonated *Toxocara* spp. eggs present in contaminated food, water, or soil. An additional route of infection involves ingesting raw or undercooked viscera/meat of mammal or bird paratenic hosts harboring infective larvae (L_3_) [3]. Following ingestion, *Toxocara* larvae from the eggs and/or tissues are released in the small intestinal lumen. They penetrate the intestinal wall and enter the circulation to be disseminated to different organs [4]. Despite usually resulting in asymptomatic chronic infection, this larval migration may cause severe disease, affecting various organs (visceral toxocariasis), eyes (ocular toxocariasis), and central nervous system (neurotoxocariasis) [5], depending on larval load, continuous reinfection, tissue distribution, and intensity of the host inflammatory response [4].

According to a recent meta-analysis, the overall global seroprevalence for toxocariasis has been estimated as 19% (95% CI: 16.6–21.4%; 62,927/265,327) [6], which is significantly higher in vulnerable socioeconomic groups [7]. Despite its high prevalence, gaps regarding serosurveys and risk factors in vulnerable populations, including homeless people, remain.

Among vulnerability factors, homelessness has emerged as an international human rights violation [8], representing a global phenomenon affecting both developed and developing countries [9, 10]. Homelessness may lead to serious health implications [11] mainly due to insufficient healthcare, inadequate nutrition, precarious living conditions, along with physical and mental illness [12]. In addition, health status may be aggravated by substance abuse and the long-term burden of chronic diseases, increasing morbidity and premature death risks compared with housed persons [13].

Among the Latin American countries, Brazil has the most unequal income distribution, with 6.5% (13.6 million people) of the nationwide population living in extreme poverty due to economic crisis and political disarray since 2014 [14]. Extreme poverty rose to an estimated 9.5% by late 2020, worsened by the COVID-19 pandemic, particularly in major urban settings [15]. Brazilian homelessness has grown around 140% since 2012, with over half (56.2%) living in southeastern Brazil, mainly in São Paulo [16].

Despite its high prevalence in vulnerable populations, toxocariasis in persons experiencing homelessness remains to be thoroughly investigated. Accordingly, the present study aims to assess the seroprevalence and risk factors associated with *Toxocara* spp. exposure in persons experiencing homelessness and shelter workers from the day-shelter providing care to this population in São Paulo city, Brazil, the city with the largest homeless population nationwide.

## Methods

### Study area

This is a cross-sectional study of the population experiencing homelessness, and shelter workers, including healthcare and assistance professionals, such as nurses, social workers, administrative personnel, cooks, and cleaning and maintenance professionals who provide care to persons who are homeless. The study was conducted in São Paulo city (23°33′1″S, 46°38′2″W), the capital of São Paulo State, southeastern Brazil, the most populous city in Latin America, with 11,253,500 habitants at the time, a high Human Development Index (HDI) (0.805), and with a humid subtropical climate and average temperatures varying from 19 °C (winter) to 25 °C (summer) [17].

Sample collection was performed at a major shelter that serves the second-highest population experiencing homelessness in São Paulo City and accounts for 4779/24,344 (19.6%) persons experiencing homelessness in this town [17]. This shelter was a day-only public service center providing three meals a day and medical assistance to persons who were experiencing homelessness, with no dormitory or sleepover permission.

### Sampling and questionnaires

Participants signed a consent form prior to sample collection. Serum samples were collected by venipuncture using commercial vacuum tubes (Vacutainer, BD Co., Curitiba, Brazil). Samples were centrifuged at 1295 × *g* for 5 min, and the separated serum was stored at −20 ºC until further testing.

The questionnaire for people experiencing homelessness collected information regarding sociodemographic aspects (city of origin, age, gender identity, self-identified race/ethnicity, and level of education), previous assistance by the government's healthcare program "Street Clinics," time being homeless, contact with dogs and cats, showering frequency, drinking water source/type, raw meat intake, contact with soil, and onychophagy.

All shelter workers were also interviewed with a structured questionnaire for sociodemographic, behavioral, and clinical information. For the shelter workers, questions were evaluated for sociodemographic aspects (city of origin, age, gender identity, self-identified race/ethnicity, and level of education), contact with dogs or cats, drinking water source/type, raw meat intake, contact with soil, and onychophagy.

### Enzyme-linked immunosorbent assay (ELISA)

Adult *T. canis* worms discharged in the feces of naturally infected puppies were collected. Adult female nematodes were exposed to 1% sodium hypochlorite for 5 min to remove surface debris, followed by washing with normal saline for 3 min. After washing, the anterior third of the worm was dissected to collect parasite eggs [18, 19].

Eggs of *T. canis* were incubated in 2% formalin for approximately 30 days at 28 °C to facilitate embryonation. Larvae were hatched and incubated at 37 °C in serum-free Eagle's medium, according to the standard protocol [20]. The culture supernatant/medium containing the *Toxocara* excretory–secretory (TES) proteins was removed at weekly intervals, and to this, 5 µl/ml of the protease inhibitor, phenylmethylsulfonyl fluoride (PMSF; 200 mM) was added. The TES proteins in the culture medium were concentrated using a commercial ultra-centrifuge filtration unit (Millipore, Danvers, MA, USA), dialyzed against distilled water, centrifuged (18,500 × *g* for 60 min at 4 °C), and filtered using 0.22 µm membrane filters (Millipore). The protein concentration of the resulting TES fraction was determined as previously described [21].

Cross-reaction with other ascarids was avoided by pre-incubating serum samples with *Ascaris suum* adult worm extract (AWE), following an established protocol [19]. Briefly, adult *A. suum* recovered from the intestine of slaughtered pigs were macerated in distilled water. To this, one part of NaOH and nine parts of water were added, making a final concentration of 0.15 M. After incubation at room temperature for 2 h, the pH of the material was neutralized with 6 M HCI and centrifuged at 18,500 × *g* for 20 min at 4 °C. After removing the lipids with ether, the final supernatant was filtered through 0.22 µm pore-sized membrane filters (Millipore) to obtain the AWE.

All serum samples used in TES-ELISA were pre-incubated for 30 min at 37 ºC with an AWE solution (25.0 µg/µl) in 0.01 M phosphate-buffered saline (PBS, pH 7.2) containing 0.05% Tween 20 (PBS-Tween) (Sigma, St. Louis, MO, USA) at a dilution of 1:200. Polystyrene 96-well microtiter plates (Corning, Costar, NY, USA) were coated with TES proteins at a concentration of 1.9 µg/µl per well using the coating buffer, 0.06 M carbonate-bicarbonate buffer, at pH 9.6. The coating was accomplished by incubating these plates for 1 h at 37 °C and then for 18 h at 4 ºC. The plates were subsequently blocked for 1 h at 37 °C with 3% skimmed milk (Molico®, Nestle, Brazil) diluted in PBS-Tween. Serum samples pre-adsorbed with AWE were added to wells in duplicate, incubated at 37 °C for 30 min, and washed three times with PBS-5% Tween. Anti-human immunoglobulin G (IgG; Fc-specific) peroxidase secondary antibody (Sigma A6029, St. Louis, MO, USA) was added at a 1:5000 dilution and incubated for 45 min at 37 °C.

After an additional washing cycle, the peroxidase substrate o-phenylenediamine (Sigma*Fast*™ OPD, Sigma P9187, St. Louis, MO, USA) was added to the wells and incubated for 5 min to visualize the antigen–antibody reaction. The reaction was stopped by adding 2 N sulfuric acid and absorbance was measured at 492 nm. Positive and negative controls were included in each plate. The cut-off value was determined as the mean absorbance of 96 negative control sera plus three standard deviations. Antibody levels were expressed as reactivity indexes (RI), which were calculated as the ratio between the absorbance value of each sample and the cut-off value.

Positive sera were titrated using the same ELISA methodology as described above [22], except that sera were diluted 1:200 in the *Ascaris* adsorbent, AWE, and incubated for 30 min at 37 °C, then twofold serial dilutions were prepared (up to 1:12.800) using blocking solution and incubated for 60 min at 37 °C. The final titer of each serum sample was the highest dilution where a positive result was obtained.

The avidity index (AI) of IgG was performed by a dissociation method, using a 6 M urea solution as the denaturing agent [23]. The AI, expressed as a percentage, was calculated as the mean optical density (OD) of (urea-treated/urea-untreated) × 100. Values of AI up to 50 were considered low avidity (indicating recently acquired infection or recent toxocariasis), and AI values exceeding 50 were considered an indicator of high avidity (past toxocariasis).

### Statistical analysis

Statistical analyses were performed using the programming language and free software environment R. Potential risk factors for toxocariasis were assessed by univariate analysis (Chi-square test or Fisher's exact test). Variables with a *P*-value ≤ 0.2 in the univariate analysis were included in the multivariate analysis.

The model was adjusted using the Hosmer–Lemeshow test. The predictive performance of the final model was assessed by measuring the area under the receiver operator characteristic (ROC) curve. To increase the accuracy of the final model, predictive variables were tested for collinearity and the presence of influential observations [24, 25].

Multivariate logistic regression was used to assess the strength of the association between the presence of anti-*Toxocara* spp. antibodies and factors associated with toxocariasis and the results were expressed as odds ratios (ORs). Associations with a *P*-value < 0.05 were considered statistically significant.

## Results

Overall, anti-*Toxocara* IgG antibodies were detected in 89/194 (45.9%, 95% CI: 39.0–52.9) persons experiencing homelessness and 22/79 (27.8%, 95% CI: 19.2–38.6) in related shelter workers. The endpoint titers ranged from 400 to 6400. Seroprevalence in persons experiencing homelessness was statistically higher (*χ*^2^ = 6.834; df = 1; OR = 2.2; 95% CI = 1.25–3.87; *P* = 0.0089) than in workers from health and social shelters by the Chi-square test.

According to the *χ*^2^ tests, associated risk factors, including gender (OR = 1.4; CI = 0.28–1.77; *P* = 0.694), race/ethnicity (OR = 1.1; CI 95 = 0.57–1.95; *P* = 0.994), previous assistance by the government’s healthcare program “Street Clinics” (OR = 0.6; 95% CI = 0.30–1.06; *P* = 0.103), homelessness time (OR = 0.78; 95% CI = 0.37–1.66; *P* = 0.349), contact with dogs (OR = 1.1; 95% CI = 0.43–2.73; *P* > 1.0), cats (OR = 0.8; CI = 0.23–2.80; *P* = 0.998) or soil (OR = 0.8; 95% CI = 0.43–1.51; *P* = 0.606), bathing frequency (OR = 0.6; 95% CI = 0.20–1.44; *P* = 0.322), availability of drinking water (OR = 2.4; 95% CI = 0.27–68.5; *P* = 0.627), and onychophagy (OR = 1.0 95% CI = 0.46–1.95; *P* > 1.0) were not statistically significant (Table 1). The only statistically significant associated risk factor for persons experiencing homelessness was having higher education, which was a protective factor against *Toxocara* spp. infection (OR = 0.2; 95% CI = 0.06–0.72; *P* = 0.018), as revealed by multivariate analysis (logistic regression) (Table 2).

**Table 1:** Bivariate analysis including the associated risk factors for anti-*Toxocara* spp. antibodies in people experiencing homeless in São Paulo city, Brazil (*N* = 194; positive = 89 and negative = 105)

Variable	Positive (%)	Negative (%)	Odds Ratio (95% CI)	Statistical analysis
City of origin				0.638
São Paulo city	30 (33.7)	31 (29.5)	Reference	
Others	59 (66.3)	74 (70.5)	1.2 (0.66–2.24)	
Gender identity				0.599
Female	8 (8.0)	13 (12.4)	Reference	
Male	81 (92.0)	92 (87.6)	1.4 (0.28–1.77)	
Age (Years)				0.112
> 60	17 (19.3)	14 (13.3)	Reference	
30 to 60	55 (62.5)	80 (76.2)	0.6 (0.25–1.26)	
< 60	16 (18.2)	11 (10.5)	1.2 (0.41–3.48)	
Educational background				0.051
Elementary school	47 (52.8)	44 (41.9)	Reference	
High school	38 (42.7)	46 (43.8)	0.8 (0.43–1.41)	
College	4 (4.5)	15 (14.3)	0.3 (0.07–0.79)	
Race/ethnicity				0.994
White	27 (30.3)	33 (31.4)	Reference	
Non-white	62 (69.7)	72 (68.6)	1.1 (0.57–1.95)	
Street clinics				0.103
No	62 (72.9)	63 (60.6)	Reference	
Yes	23 (27.1)	41 (39.4)	0.6 (0.30–1.06)	
Years of homelessness				0.349
< 1 year	23 (34.8)	26 (33.8)	Reference	
1–10 years	27 (40.9)	39 (50.6)	0.78 (0.37–1.66)	
> 10 years	16 (24.2)	12 (15.6)	1.5 (0.58–3.91)	
Drinking water				0.627
No	1 (1.18)	3 (2.97)	Reference	
Yes	84 (98.8)	98 (97.0)	2.4 **(**0.27–68.5)	
Raw meat intake				0.228
No	76 (90.5)	85 (83.3)	Reference	
Yes	8 (9.52)	17 (16.7)	0.5 (0.20–1.28)	
Contact with dog				1.0
No	79 (88.8)	94 (89.5)	Reference	
Yes	10 (11.2)	11 (10.5)	1.1 (0.43–2.73)	
Contact with cat				0.998
No	84 (94.4)	98 (93.3)	Reference	
Yes	5 (5.62)	7 (6.67)	0.8 (0.23–2.80)	
Contact with soil				0.606
No	64 (72.7)	71 (68.3)	Reference	
Yes	24 (27.3)	33 (31.7)	0.8 (0.43–1.51)	
Onychophagy				1.0
No	69 (80.2)	81 (79.4)	Reference	
Yes	17 (19.8)	21 (20.6)	1.0 (0.46–1.95)	
Showering				0.322
Daily	82 (92.1)	91 (86.)	Reference	
Rarely	7 (7.87)	14 (13.3)	0.6 (0.20–1.44)

**Table 2:** Multivariate analysis (logistic regression) including risk factors for anti-*Toxocara* spp. antibodies in people experiencing homeless in São Paulo city, Brazil (*N* = 194; positive = 89 and negative = 105)

Variable	Positive (%)	Negative (%)	Odds ratio (95% CI)	Statistical analysis
Age (Years)				
> 60	17 (19.3)	14 (13.3)	Reference	
30–60	55 (62.5)	80 (76.2)	0.6 (0.27–1.46)	0.280
< 60	16 (18.2)	11 (10.5)	1.1 (0.38–3.48)	0.816
Educational background				
Elementary school	47 (52.8)	44 (41.9)	Reference	
High school	38 (42.7)	46 (43.8)	0.6 (0.34–1.20)	0.165
College	4 (4.5)	15 (14.3)	0.2 (0.06–0.72)	0.018
Street clinics				
No	62 (72.9)	63 (60.6)	Reference	
Yes	23 (27.1)	41 (39.4)	0.5 (0.28–1.03)	0.066

Persons experiencing homelessness sampled herein were mainly 173/194 (89.2%) men, of which 81/173 (46.8%) were seropositive, whereas 21/194 (10.8%) were women and 8/21 (38.0%) seropositive. Mainly, 135/194 (69.6%) persons experiencing homelessness were between 30 and 60 years old, 133/194 (68.6%) were from another city than São Paulo, of which 59/133 (44.4%) were seropositive, while 61/194 (31.4%) were born in São Paulo and 30/61 (49.2%) seropositive. No significant differences were found between these groups.

Based on available health records at the shelter, 11/194 (5.7%) persons experiencing homelessness were seropositive to HIV, and 29/194 (14.9%) had syphilis. Anti-*Toxocara* antibodies were observed in 2/11 (18.2%) HIV and 14/29 (48.3%) syphilis-positive individuals. No statistical association was revealed when comparing the presence of anti-*Toxocara* antibodies and HIV *χ*^2^ = 3.51 df = 1; OR: 0.22; CI 95% = 0.05–1.0; *P* = 0.072) or syphilis (*χ*^2^ = 0.28; df = 1; OR: 0.74; CI 95% = 0.38–1.65; *P* = 0.597) seropositivity, by *χ*^2^ test.

No risk factor was associated with seropositivity of anti-*Toxocara* spp. antibodies and shelter workers, according to the statistical analysis (Additional file 1: Table S1).

## Discussion

To the authors' knowledge, this is the first study conducting a serosurvey to detect anti-*Toxocara* spp. antibodies and the associated risk factors among persons experiencing homelessness, and revealed a high seroprevalence for anti-*Toxocara* IgG (45.9%; CI 95%: 39.0–52.9%) in adults experiencing homelessness in São Paulo. Our study has also shown that persons experiencing homelessness were 2.2 times more likely to be infected than shelter workers, demonstrating a difference in toxocariasis exposure. In Brazil, the seroprevalence of toxocariasis has been widely reported, ranging from 4.2% [26] to 63.6% [27] in children, and from 8.7% [28] to 71.8% [29] in adult populations. Recently, 58/280 (20.7%) pregnant women [30] and 212/328 (64.6%) inhabitants of a Brazilian traditional seashore population were seropositive for anti-*Toxocara* antibodies [31].

Although 45.9% frequency herein has been substantially higher than the overall 27.5% (CI 95%: 14.8–42.3%) seroprevalence for toxocariasis in Brazil, results were similar to 141/306 (46.3%) adult blood donors living in the most populated city of northeastern Brazil [6], indicating a multifactorial cause for toxocariasis, as previously observed [32]. Nonetheless, the present study has assessed a highly vulnerable population, which has been associated with high risks of disease and premature death [13]. Thus, homeless living conditions may have influenced the high prevalence observed here compared with domiciled shelter workers.

Although several risk factors have been reportedly associated with toxocariasis, including being male, youngster, having contact with dogs, cats, soil, consuming raw meat, and drinking untreated water [6], none was statistically associated with the presence of anti-*Toxocara* antibodies. Such outcomes may be due to low sampling or high sample heterogeneity, or more likely, the skewness of the homeless population, mostly males, adults, and reportedly drug users [33, 34]. Not surprisingly, the predominance of males in the homeless population observed here has already been reported in Canada (273/455; 60%) [35], Nicaragua (62/82; 75.7) (9), and Brazil (635/701; 90.7%) [36]. As the population studied here primarily represented adults between 30 and 60 years old, youth could not be adequately tested as an associated risk factor for toxocariasis.

Besides being the largest city in Latin America, São Paulo has been among the most multicultural cities worldwide, accounting for the largest population of migrants, immigrants, and refugees nationwide [37]. Such a scenario of social vulnerability has challenged health authorities, for example, seropositivity of anti-*Treponema pallidum* antibodies has been significantly higher (*P* = 0.043) in immigrant persons experiencing homeless conditions [38]. Fortunately, although the homeless population was predominantly composed of 133/194 (68.6%) migrants and 134/194 (69.1%) non-white persons, both variables were not associated with toxocariasis.

Although pet ownership was not identified as an associated risk factor for toxocariasis, dogs and cats have been well established as the primary animal hosts for *Toxocara* spp., particularly in developing countries where most cats and dogs have access to public parks and playgrounds, leading to soil contamination and human exposure to infective eggs [7]. Even though pet ownership, especially of dogs, has been reported among persons experiencing homelessness [39], in this study, only 21/194 (10.8%) individuals reported contact with dogs and 12/194 (6.2%) with cats. Interestingly, only 57/194 (29.4%) persons experiencing homelessness referred to having direct contact with soil, probably because their living areas within the eastern-urban setting of São Paulo city were covered mainly by concrete, asphalt, and/or cement.

As a limitation to the One Health approach, the study herein has not surveyed dog feces and soil for the presence of *Toxocara* spp. eggs. Nevertheless, the lack of statistical significance of dog and cat ownership and soil contact, combined with high seropositivity to toxocariasis in persons who are homeless, may indicate high environmental exposure to infection. Thus, animal health interventions, including scooping pet feces and deworming dogs and cats (as well as all other owned and stray pets citywide), should be considered to mitigate the risk of environmental contamination by *Toxocara* spp.

In addition to environmental contamination, the ingestion of raw or undercooked meat or the viscera of paratenic hosts, including cows, pigs, and chickens, has been considered important risk factors for toxocariasis [40–42]. In this study, only 25/194 (12.8%) homeless persons referred to ingesting raw meat, corroborating previous studies that indicated that access to fresh meat, fish, vegetables, and fruits by persons experiencing homelessness was limited due to poverty conditions [43, 44]. Moreover, our research group has shown that persons experiencing homelessness in the same city of São Paulo were less likely to be infected by *Toxoplasma gondii* mainly due to consuming processed and ready-to-eat foods [34]. Thus, similar to toxoplasmosis, ingesting raw meat may represent a less important transmission route of toxocariasis for individuals who are homeless.

The present study revealed that having a college degree was a protective factor for *Toxocara* infection (OR: 0.23; *P* = 0.018), corroborating that educational level has been a social determinant for human toxocariasis [6]. As previously shown, individuals with only high school education were more likely to be infected (OR = 1.54) when compared with those with a college degree [45], and toxocariasis frequency was significantly higher in persons missing a high school degree [46, 47]. In addition to health and self-hygiene access and awareness, persons who are homeless and hold a college degree may have lived in better socioeconomic conditions before being houseless, reducing the exposure period to toxocariasis.

The occurrence of HIV (5.7%) and syphilis (14.9%) in the studied population corroborate other studies focused on high-risk sexually transmitted infections in persons experiencing homelessness [48, 49]; however, their presence was not associated with seropositivity for *Toxocara* antibodies. A previous study has shown that being under treatment for HIV was significantly associated with toxocariasis (*P* = 0.0087), and co-infection assessment was crucial to establish the synergism between HIV and tissue helminths [50].

Ethnic and racial disparities have also been associated with discrimination of persons experiencing homelessness [51], as these people have been more frequently associated with Black ethnicity [52]. Again, although high toxocariasis seropositivity has been linked to Black, non-Hispanics, and other ethnic groups [44], 134/194 (69,1%) persons experiencing homelessness herein identified themselves as Black, and no statistical significance was found.

As a limitation, despite being considered the most widely employed test in toxocariasis serosurveys and diagnosis, the ELISA test has failed to differentiate between recent and chronic infection [53]. Here, IgG avidity was assessed to distinguish recent from past toxocariasis [23], and the avidity index indicated that all ELISA-positive individuals had a past infection (high avidity > 50). Thus, the presence of anti-*Toxocara* antibodies was independent of the duration of homelessness. Another limitation in this study includes the possibility of biased memory precision given by the individuals responding to the sociodemographic questionnaire, where no precise inference could be made on the homelessness time frame. Nevertheless, 94/143 (65.7%) persons experiencing homelessness have declared to have been living for more than one year under homelessness conditions, supporting the long-term infection as detected by the avidity index.

This study has also been limited by the difficulty in accessing individuals who were homeless, partially explained by the lack of studies involving such populations worldwide, mainly due to refusal to answer the sociodemographic questionnaire and blood sampling. Although such limitation may have impaired reliable outcome data to provide robust statistical analysis, the results have contributed to our understanding of toxocariasis in the homeless population.

Finally, questionnaire information to assess persons experiencing homelessness may be problematic, particularly regarding food intake and dietary habits, once such a population has often shown a chaotic lifestyle and a high prevalence of drug abuse and mental health disorders. Further studies should be conducted using higher sampling numbers and from different homeless populations worldwide to establish the exact impact of toxocariasis in such populations.

## Conclusions

This is the first study reporting a serosurvey of *Toxocara* spp. antibodies in persons experiencing homelessness. Despite the limitations, our findings indicated that the frequency of anti-*Toxocara* antibodies in persons experiencing homelessness was relatively higher compared with other populations. Besides educational level as a protective factor for toxocariasis, no other risk factor was associated with *Toxocara* spp. exposure in persons experiencing homelessness.

## Supplementary Information

**Additional file 1: Table S1**. Associated risk factors for toxocariasis in shelter workers of São Paulo city, Brazil (N=79).

## Data Availability

The datasets used and/or analyzed during the current study are available from the corresponding author on reasonable request.

## Abbreviations

HIV: Human immunodeficiency virus
AWE: Adult worm extract
ELISA: Enzyme-linked immunosorbent assay
PBS: Phosphate-buffered saline

## References

[1] CDC—Toxocariasis. 2022. https://www.cdc.gov/parasites/toxocariasis/index.html. Accessed 30 Aug 2022.

[2] Bowman DD (2020). History of Toxocara and the associated larva migrans. Adv Parasitol.

[3] Ma G, Holland CV, Wang T, Hofmann A, Fan C-K, Maizels RM (2018). Human toxocariasis. Lancet Infect Dis.

[4] Despommier D (2003). Toxocariasis: clinical aspects, epidemiology, medical ecology, and molecular aspects. Clin Microbiol Rev.

[5] Hotez PJ (2020). Toxocariasis: a neglected infection for the Anthropocene epoch. Adv Parasitol.

[6] Rostami A, Riahi SM, Holland CV, Taghipour A, Khalili-Fomeshi M, Fakhri Y (2019). Seroprevalence estimates for toxocariasis in people worldwide: a systematic review and meta-analysis. PLoS Negl Trop Dis.

[7] Chen J, Liu Q, Liu G-H, Zheng W-B, Hong S-J, Sugiyama H (2018). Toxocariasis: a silent threat with a progressive public health impact. Infect Dis Poverty.

[8] OHCHR|Homelessness and human rights. https://www.ohchr.org/en/issues/housing/pages/homelessnessandhumanrights.aspx. Accessed 30 Aug 2022.

[9] Vázquez JJ, Berríos AE, Bonilla E, Suarez AC (2019). Homeless people in León (Nicaragua): conceptualizing and measuring homelessness in a developing country. Am J Orthopsychiatry.

[10] Vázquez JJ, Berríos A (2022). Unhappiness and casual attributions of homelessness among people living homeless in León (Nicaragua). J Community Psychol.

[11] Fransham M, Dorling D (2018). Homelessness and public health. BMJ (Clinical research ed.).

[12] Lancet T (2020). Redefining vulnerability in the era of COVID-19. Lancet (London, England)..

[13] Omerov P, Craftman ÅG, Mattsson E, Klarare A (2020). Homeless persons’ experiences of health- and social care: a systematic integrative review. Health Soc Care Community.

[14] Economic Commission for Latin America and the Caribbean. https://www.cepal.org/en. Accessed 30 Aug 2022.

[15] COVID-19 Dominated Global Health in 2021. Will 2022 Be the Same? https://unfoundation.org/blog/post/covid-19-dominated-global-health-in-2021-will-2022-be-the-same/?gclid=Cj0KCQiA64GRBhCZARIsAHOLriIXNIOFYV3FLNl-svqgfQAdgrOUSigmqqZ4coMJhPiUYHQ6AFo0SxMaAr5OEALw_wcB Accessed 30 Aug 2022.

[16] Nota Técnica—2020—Junho—Número 73—Disoc—Estimativa da população em situação de rua no Brasil (setembro de 2012 a março de 2020). https://www.ipea.gov.br/portal/index.php?option=com_content&view=article&id=35812 Accessed 30 Aug 2022.

[17] São Paulo (SP) | Cidades e Estados | IBGE. https://www.ibge.gov.br/cidades-e-estados/sp/sao-paulo.html Accessed 30 Aug 2022.

[18] de Savigny DH, Voller A, Woodruff AW (1979). Toxocariasis: serological diagnosis by enzyme immunoassay. J Clin Pathol.

[19] Elefant GR, Shimizu SH, Arroyo Sanchez MC, Abe Jacob CM, Ferreira AW (2006). A serological follow-up of toxocariasis patients after chemotherapy based on the detection of IgG, IgA, and IgE antibodies by enzyme-linked immunosorbent assay. J Clin Lab Anal.

[20] Elefant GR, Shimizu SH, Sanchez MCA, Jacob CMA, Ferreira AW (2006). A serological follow-up of Toxocariasis patients after chemotherapy based on the detection of IgG, IgA, and IgE antibodies by enzyme-linked immunosorbent assay. J Clin Lab Anal.

[21] Lowry OH, Rosebrough NJ, Farr AL, Randall RJ (1951). Protein measurement with the Folin phenol reagent. J Biol Chem.

[22] Pereira LC, Elefant GR, Nóbrega YM, Vital T, Nitz N, Gandolfi L (2016). *Toxocara* spp. seroprevalence in pregnant women in Brasília, Brazil. Rev Soc Bras Med Trop.

[23] Dziemian E, Zarnowska H, Kołodziej-Sobocińska M, Machnicka B (2008). Determination of the relative avidity of the specific IgG antibodies in human toxocariasis. Parasite Immunol.

[24] Robin X, Turck N, Hainard A, Tiberti N, Lisacek F, Sanchez J-C (2011). pROC: an open-source package for R and S+ to analyze and compare ROC curves. BMC Bioinform.

[25] Subirana I, Sanz H, Vila J (2014). Building bivariate tables: the compare groups package for R. J Stat Softw [Internet].

[26] Guilherme EV, Marchioro AA, Araujo SM, Falavigna DLM, Adami C, Falavigna-Guilherme G (2013). Toxocariasis in children attending a Public Health Service Pneumology Unit in Paraná State, Brazil. Rev Inst Med Trop Sao Paulo.

[27] Silva MB, Amor ALM, Santos LN, Galvão AA, Oviedo Vera AV, Silva ES (2017). Risk factors for *Toxocara* spp. seroprevalence and its association with atopy and asthma phenotypes in school-age children in a small town and semi-rural areas of Northeast Brazil. Acta Trop.

[28] Negri EC, Santarém VA, Rubinsky-Elefant G, Giuffrida R (2013). Anti-*Toxocara* spp. antibodies in an adult healthy population: serosurvey and risk factors in Southeast Brazil. Asian Pac J Trop Biomed.

[29] Araújo AC, Villela MM, Sena-Lopes Â, da Farias NAR, de Faria LMJ, da Avila LFC (2018). Seroprevalence of Toxoplasma gondii and Toxocara canis in a human rural population of Southern Rio Grande do Sul. Rev Inst Med Trop Sao Paulo.

[30] de Oliveira AP, Lescano SZ, Giuffrida R, Kmetiuk LB, Dos Santos AP, Dangoudoubiyam S (2021). Serosurvey of anti-Toxocara antibodies and risk factors in adolescent and adult pregnant women of southeastern Brazil. PLoS Negl Trop Dis.

[31] Delai RR, Freitas AR, Kmetiuk LB, Merigueti YFFB, Ferreira IB, Lescano SAZ (2021). One Health approach on human seroprevalence of anti-Toxocara antibodies, *Toxocara* spp. eggs in dogs and sand samples between seashore mainland and island areas of southern Brazil. One Heal [Internet]..

[32] Holland CV (2017). Knowledge gaps in the epidemiology of Toxocara: the enigma remains. Parasitology.

[33] Do Couto AC, Kmetiuk LB, Delai RR, Brandão APD, Monteiro CO, da Silva LHA (2021). High SARS-CoV-2 seroprevalence in persons experiencing homelessness and shelter workers from a day-shelter in São Paulo. Brazil. PLoS Negl Trop Dis..

[34] Felipetto LG, Teider-Junior PI, Da Silva FFV, Yamakawa AC, Kmetiuk LB, Do Couto AC (2020). Serosurvey of anti-toxoplasma gondii antibodies in homeless persons of São Paulo City, Southeastern Brazil. Front Public Heal.

[35] Gentil L, Grenier G, Bamvita J-M, Dorvil H, Fleury M-J (2019). Profiles of quality of life in a homeless population. Front Psychiatry.

[36] Hungaro AA, Gavioli A, Christóphoro R, Marangoni SR, Altrão RF, Rodrigues AL (2020). Homeless population: characterization and contextualization by census research. Rev Bras Enferm.

[37] de Oliveira MA, de Boska GA, de Oliveira MAF, Barbosa GC (2021). Access to health care for people experiencing homelessness on Avenida Paulista: barriers and perceptions. Rev da Esc Enferm da USP..

[38] Felipetto LG, Teider-Junior PI, da Silva FFV, do Couto AC, Kmetiuk LB, Martins CM (2021). Serosurvey of anti-treponema pallidum (syphilis), anti-hepatitis C virus and anti-HIV antibodies in homeless persons of São Paulo city, southeastern Brazil. Braz J Infect Dis Off Publ Braz Soc Infect Dis..

[39] Scanlon L, Hobson-West P, Cobb K, McBride A, Stavisky J (2021). Assessment of health and welfare in a small sample of dogs owned by people who are homeless. Vet Rec.

[40] Morimatsu Y, Akao N, Akiyoshi H, Kawazu T, Okabe Y, Aizawa H (2006). A familial case of visceral larva migrans after ingestion of raw chicken livers: appearance of specific antibody in bronchoalveolar lavage fluid of the patients. Am J Trop Med Hyg.

[41] Choi D, Lim JH, Choi DC, Lee KS, Paik SW, Kim SH (2012). Transmission of Toxocara canis via ingestion of raw cow liver: a cross-sectional study in healthy adults. Korean J Parasitol.

[42] Song HB, Lee D, Jin Y, Kang J, Cho S-H, Park MS (2020). Prevalence of Toxocariasis and its risk factors in patients with eosinophilia in Korea. Korean J Parasitol.

[43] Li A, Dachner N, Tarasuk V (2009). Food intake patterns of homeless youth in Toronto. Can J Public Health.

[44] Fallaize R, Seale JV, Mortin C, Armstrong L, Lovegrove JA (2017). Dietary intake, nutritional status and mental wellbeing of homeless adults in Reading, UK. Br J Nutr.

[45] Walsh MG, Haseeb MA (2014). Small-area estimation of the probability of Toxocariasis in New York City based on sociodemographic neighborhood composition. PLoS ONE.

[46] Erickson LD, Gale SD, Berrett A, Brown BL, Hedges DW (2015). Association between toxocariasis and cognitive function in young to middle-aged adults. Folia Parasitol.

[47] Berrett AN, Erickson LD, Gale SD, Stone A, Brown BL, Hedges DW (2017). Toxocara seroprevalence and associated risk factors in the United States. Am J Trop Med Hyg.

[48] Fazel S, Geddes JR, Kushel M (2014). The health of homeless people in high-income countries: descriptive epidemiology, health consequences, and clinical and policy recommendations. Lancet.

[49] Caccamo A, Kachur R, Williams SP (2017). Narrative review: sexually transmitted diseases and homeless youth—what do we know about sexually transmitted disease prevalence and risk?. Sex Transm Dis.

[50] Noormahomed EV, Nhacupe N, Mascaró-Lazcano C, Mauaie MN, Buene T, Funzamo CA (2014). A cross-sectional serological study of cysticercosis, schistosomiasis, toxocariasis and echinococcosis in HIV-1 infected people in Beira, Mozambique. PLoS Negl Trop Dis.

[51] Wrighting Q, Reitzel LR, Chen T-A, Kendzor DE, Hernandez DC, Obasi EM (2019). Characterizing discrimination experiences by race among homeless adults. Am J Health Behav.

[52] Baggett TP, Keyes H, Sporn N, Gaeta JM (2020). Prevalence of SARS-CoV-2 infection in residents of a large homeless shelter in Boston. JAMA J Am Med Assoc.

[53] Noordin R, Yunus MH, Tan Farrizam SN, Arifin N (2020). Serodiagnostic methods for diagnosing larval toxocariasis. Adv Parasitol England.

