# Supplementary material for: Serosurvey of anti-Toxocara canis antibodies in people experiencing homelessness and shelter workers from São Paulo, Brazil
Source: Parasit Vectors. 2022 Oct 17;15:373. doi: 10.1186/s13071-022-05499-x (PMC9574839; doi:10.1186/s13071-022-05499-x)
Supplement: Supplementary file 1 — Additional file 1: Table S1. Associated risk factors for toxocariasis in shelter workers of São Paulo city, Brazil (N=79). [file 13071_2022_5499_MOESM1_ESM.docx]

**Additional file 1 Table S1** Bivariate analysis including the associated risk factors for anti-*Toxocara* spp. antibodies in shelter workers of São Paulo city, Brazil (N = 79; positive= 22 and negative= 57)

| Variable | Positive (%) | Negative (%) | Odds Ratio  (95% CI) | *P* value |
| --- | --- | --- | --- | --- |
| City of origin* |  |  |  | 1.0 |
| São Paulo city | 13 (59.1) | 34 (61.8) | Reference |  |
| Others | 9 (49.9) | 21 (38.2) | 1.1 (0.39-1.10) |  |
| Gender |  |  |  | 0.364 |
| Female | 8 (36.4) | 29 (50.9) | Reference |  |
| Male | 14 (63.6) | 28 (49.1) | 1.8 (0.65-5.18) |  |
| Age (Years) |  |  |  | 0.887 |
| > 60 | 1 (4.6) | 2 (3.5) | Reference |  |
| 30 to 60 | 17 (77.3) | 46 (80.7) | 0.7 (0.05-23.0) |  |
| < 30 | 4 (18.2) | 9 (15.8) | 0.9 (0.05-32.5) |  |
| Educational background |  |  |  | 0.154 |
| Elementary school | 13 (59.1) | 21 (36.8) | Reference |  |
| High school | 2 (9.1) | 5 (8.8) | 0.7 (0.08-3.85) |  |
| College | 7 (31.8) | 31 (54.4) | 0.4 (0.12-1.08) |  |
| Race/ Ethnicity* |  |  |  | 0.793 |
| White | 9 (40.9) | 19 (34.5) | Reference |  |
| Non-white | 13 (59.1) | 36 (65.5) | 0.8 (0.27- 2.18) |  |
| Drinking water* |  |  |  | 1.0 |
| No | 1 (5.3) | 2 (4.3) | Reference |  |
| Yes | 18 (94.7) | 45 (95.7) | 0.8 (0.06-24.8) |  |
| Raw meat intake* |  |  |  | 1.0 |
| No | 10 (90.9) | 28 (87.5) | Reference |  |
| Yes | 1 (9.1) | 4 (12.5) | 0.8 (0.03-6.43) |  |
| Contact with dog* |  |  |  | 0.074 |
| No | 9 (42.9) | 37 (68.5) | Reference |  |
| Yes | 12 (57.1) | 17 (31.5) | 2.9 (1.01-8.37) |  |
| Contact with cat* |  |  |  | 0.196 |
| No | 15 (71.4) | 46 (85.2) | Reference |  |
| Yes | 6 (28.6) | 8 (14.8) | 2.2 (0.64-7.82) |  |
| Contact with soil* |  |  |  | 0.065 |
| No | 15 (71.4) | 48 (90.6) | Reference |  |
| Yes | 6 (28.6) | 5 (9.4) | 3.7 (0.97-15.2) |  |
| Onychophagy* |  |  |  | 0.497 |
| No | 16 (76.2) | 46 (85.2) | Reference |  |
| Yes | 5 (23.8) | 8 (14.8) | 1.8 (0.47-6.36) |  |

*Missing information: city of origin = 2.53%; race/ ethnicity= 2.53%; contact with dog = 5.06%; contact with cat= 5.06%; onychophagy= 5.06%; contact with soil= 6.33%; drinking water= 16.46%; raw meat intake= 45.57%. None of tested variables was significant at the bivariate model. However, variables with significance lower than 0.20 and missing less than 10% were included at the multivariate model (educational background, contact with dog, contact with cat, and contact with soil).

**Additional file 1 Table S2** Multivariate analysis (logistic regression) including risk factors for anti-*Toxocara* spp. antibodies in shelter workers of São Paulo city, Brazil (N = 79; positive= 22 and negative= 57)

| Variable | Positive (%) | Negative (%) | Odds Ratio  (95% CI) | *P* value |
| --- | --- | --- | --- | --- |
| Educational background |  |  |  |  |
| Elementary school | 13 (59.1) | 21 (36.8) | Reference |  |
| High school | 2 (9.1) | 5 (8.8) | 1.0 (0.11-6.45) | 0.971 |
| College | 7 (31.8) | 31 (54.4) | 0.3 (0.09-1.07) | 0.071 |
| Contact with dog |  |  |  |  |
| No | 9 (42.9) | 37 (68.5) | Reference |  |
| Yes | 12 (57.1) | 17 (31.5) | 2.3 (0.75-7.28) | 0.147 |
| Contact with cat |  |  |  |  |
| No | 15 (71.4) | 46 (85.2) | Reference |  |
| Yes | 6 (28.6) | 8 (14.8) | 2.7 (0.66-11.58) | 0.163 |
| Contact with soil |  |  |  |  |
| No | 15 (71.4) | 48 (90.6) | Reference |  |
| Yes | 6 (28.6) | 5 (9.4) | 3.9 (0.94-17.8) | 0.063 |
